# Limitations and potential of immunotherapy in ovarian cancer

**DOI:** 10.3389/fimmu.2023.1292166

**Published:** 2024-01-09

**Authors:** Sandeep Kumar, Sayanti Acharya, Mahalakshmi Karthikeyan, Priyobrata Biswas, Sudha Kumari

**Affiliations:** Department of Microbiology and Cell Biology, Indian Institute of Science, Bangalore, India

**Keywords:** ovarian cancer, immunotherapy, immunosuppression, T cells, checkpoint inhibitors

## Abstract

Ovarian cancer (OC) is the third most common gynecological cancer and alone has an emergence rate of approximately 308,069 cases worldwide (2020) with dire survival rates. To put it into perspective, the mortality rate of OC is three times higher than that of breast cancer and it is predicted to only increase significantly by 2040. The primary reasons for such a high rate are that the physical symptoms of OC are detectable only during the advanced phase of the disease when resistance to chemotherapies is high and around 80% of the patients that do indeed respond to chemotherapy initially, show a poor prognosis subsequently. This highlights a pressing need to develop new and effective therapies to tackle advanced OC to improve prognosis and patient survival. A major advance in this direction is the emergence of combination immunotherapeutic methods to boost CD8^+^ T cell function to tackle OC. In this perspective, we discuss our view of the current state of some of the combination immunotherapies in the treatment of advanced OC, their limitations, and potential approaches toward a safer and more effective response.

## Introduction

Cancer immunotherapy has revolutionized treatment for several malignancies (1). Tumor development involves numerous immune evasion strategies of the tumor cells including the establishment of the immunosuppressive microenvironment that inhibits the anti-tumor function of the immune system (2, 3). The immunosuppressive pathways include inhibition of antigen presentation processes and cells (4), poor infiltration and activation of cytotoxic lymphocytes (5), and metabolic reprogramming of the tumor as well as immune cells reinforcing the immunosuppressive niche within the tumor (5). Immunotherapies leverage the immune system, particularly the CD8^+^ T cells, to counteract the immune evasion and immunosuppressive mechanisms in cancer. In the case of OC, the complex OC-tumor microenvironment (TME) poses a potent suppression program against the host immune system allowing tumor immune evasion and limiting the efficacy of the immunotherapeutic approaches (6).

**Figure 1:**
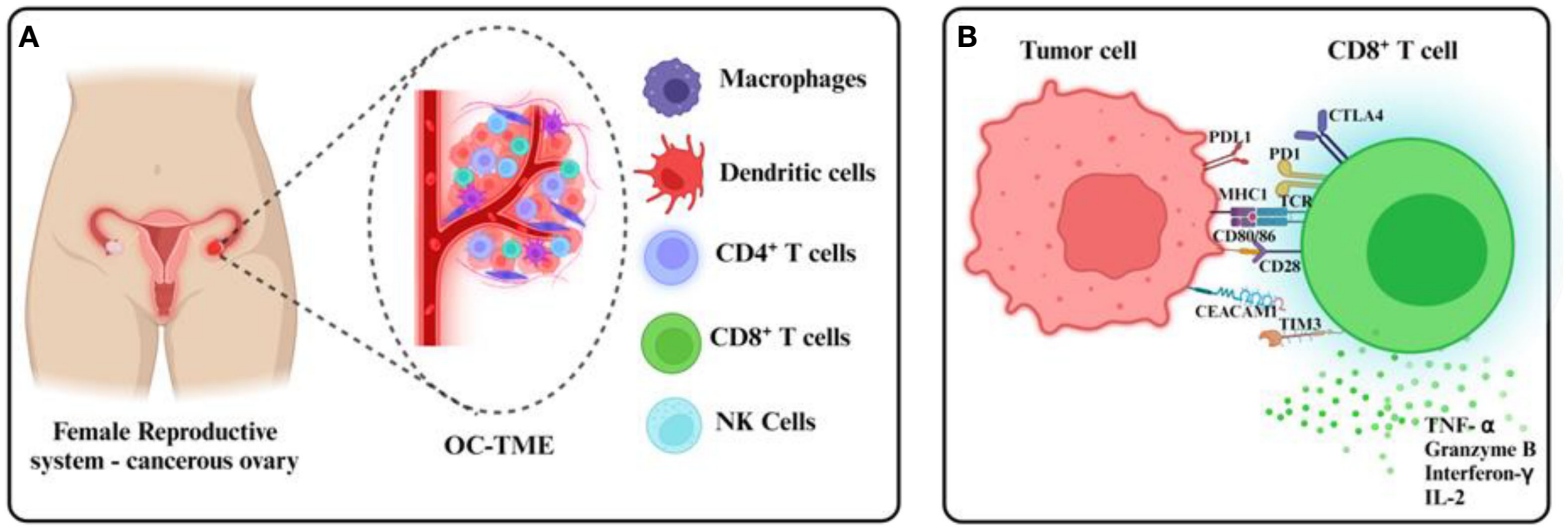
Ovarian cancer TMEs and tumor CD8+ T cell evasion. **(A)** The ovarian cancer TME comprises immune cellular components within and around the primary tumor in malignant ascites, found usually in dysfunctional states. **(B)** Immunosuppressive mechanisms including the expression of checkpoint receptors at the CD8+ T cell surface inhibit tumor recognition and cytotoxicity usually mediated by the release of cytolytic granules and effector cytokines.

There are at least two TMEs in OC reinforcing a multilayered immunosuppressive niche: the primary solid tumor and the liquid microenvironment surrounding it called malignant ascites that accumulates with the progression of the disease. Malignant ascites accumulation is one of the hallmarks of OC and it is associated with greater tumor burden, enhanced metastasis to distant organs, and more frequent recurrence of the disease (7). OC cells that are shed from the primary tumor spend most of their lifetime in the ascites microenvironment. OC conditioning of the malignant ascites further provides growth and survival advantages to the cancer cells (8, 9). Though the ascites microenvironment and peritoneal space in general is replete with numerous supposedly anti-tumor immune cells like macrophages, neutrophils, lymphocytes, NK cells, etc. (8, 10, 11), these cells are often dysfunctional and are unable to execute their anti-tumor activity (12–14) (**Figure 1A**).

## Immunosuppression in OC

Various factors in the ovarian tumor microenvironment contribute to the creation of an immunosuppressive milieu. These include both soluble factors as well as factors presented on the cells within the tumor microenvironment. Out of the soluble factors, IL10 is an important anti-inflammatory cytokine that is markedly increased in ascites from OC patients and helps in cancer cell migration and proliferation. In addition to inhibiting CD8+ T cells directly, IL10 activates STAT3 signaling in tumor cells, which promotes cell proliferation correlating with enhanced OC growth and chemoresistance (15–17). The Vascular Endothelial Growth factors (VEGFs) secreted in the ascites, particularly VEGF-A and VEGF-C, have received significant attention as the drivers of immunosuppression. Their levels are markedly elevated in malignant ascites compared to ascites of non-malignant origin. Higher VEGF levels enhance vascular permeability, secretion of matrix metalloproteinases, and angiogenesis, thereby promoting metastasis (18). Damage-Associated Molecular Patterns (DAMPs) are yet other acellular factors mediating immunosuppression. The release of DAMPs activates TME neutrophils and promotes metastasis of OC, although the cellular mechanisms enabling this effect are not fully understood. One potential mechanism is via mitochondrial DAMPs (mtDAMPs) that activate neutrophils triggering the generation of extracellular traps (NETs). Nets can subsequently cause cancer-associated thrombosis and enhanced metastasis. The mtDAMPS also serve as an important prognostic biomarker in OC associated with poor survival rates (19).

**Figure 2:**
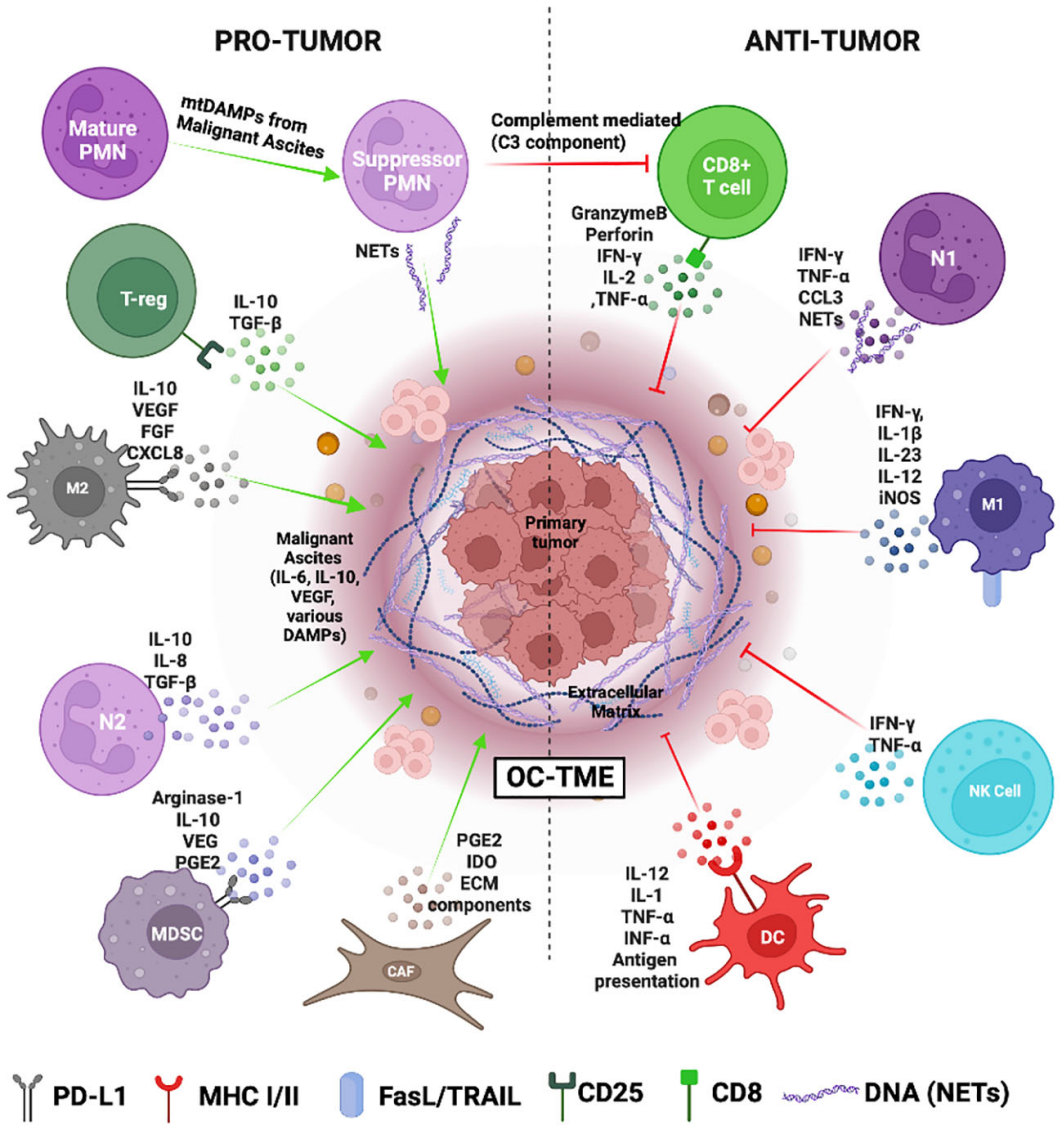
The Ovarian cancer tumor microenvironment (OC-TME). The pro- and anti-tumor function of the immune system in cancer is mediated by diverse immune cell populations that form a complex regulatory network in and around the primary tumor.

At a molecular level, the expression of immune checkpoint receptors (PD-1, CTLA4, LAG3) on T cells and their ligands (PD-L1, CD80/86, FGL1) in cellular TME leads to T cell exhaustion facilitating tumor immune evasion (20) (**Figure 1B**). Immunosuppression is further reinforced by the network of immunosuppressive cells such as the regulatory T cells (T-regs), tumor-associated macrophages (TAMs), tumor-entrained neutrophils, and myeloid–derived suppressor cells (MDSCs)- all of which suppress T cell responses (**Figure 2**; **Table 1**). In advanced cancers, there is also a ‘myeloid bias’ that skews the differentiation of immature myeloid progenitors towards granulocyte-like neutrophils (10). Classically, the Tumor-associated neutrophils (TAN) can exhibit functional polarization either into N1 (anti-tumorigenic) or N2 (pro-tumorigenic) neutrophils in response to cytokine stimulation (21, 22), and the mature circulating neutrophils are not intrinsically suppressive. However, in the case of OC, the mature circulating neutrophils acquire a suppressive phenotype once they are recruited in the ascites microenvironment, and suppress CD8+ T cells by mechanisms distinct from the classical pro-tumor N2 TANs (10). The malignant ascites-entrained suppressive neutrophils in OC although inhibit the proliferation and expansion of CTLs, they do not induce exhaustion of the CTLs. Their suppressive effect is partly mediated by the complement pathway, as inhibition of the C3 component of the complement pathway abrogates the suppressive phenotype (10, 23). Indeed, malignant ascites contains elevated amounts of the C3a component of the complement pathway (24). In addition, the formation of NETs contributes to the establishment of the pre-metastatic niche and subsequent colonization of NET-bound OC cells in the omentum (25), indicating a crucial pro-tumor role of these neutrophils in OC.

**Table 1:** Cells and their function within the OC-TME.

Immune cell type	Function	Modulation of activity	Scope for therapy	Reference
Regulatory T cells (Tregs)	Immunosuppression	CD39, perforin-granzymes mediate effector T cell inhibition, CTLA4 inhibits APC activation	Anti-CCR4 antibody	(38–40)
Tumour Associated Macrophages (TAM)-(M2)	Anti-inflammatory, Pro-tumorigenic	Polarization of M0 to M2 phenotype	Anti CSF-1R therapy, Combination therapy with angiogenic inhibitors	(41)
Myeloid-derived suppressor cells (MDSC)	Immune suppression	Arginase-1, IL-10, VEGF	Anti-CXCR2 antibody	(42, 43)
Cancer-Associated Fibroblast (CAF)	Immune suppression, over-expression of extracellular matrix proteins	Inhibition of T-cell infiltration via ECM overexpression, production of TGF-β	CAF targeted vaccines	(44)
CD8^+^ T cells (Cytotoxic T cells)	Cytotoxicity and effector immune response	down-regulation of GranzymeB, Perforin, PD-1, LAG3, TIM3, TIGIT	Immune checkpoint therapy	(45, 46)
M1 macrophage	Pro-inflammatory, anti-tumor	Polarization to M2 phenotype	Anti-CSF-1R therapy	(47)
Natural Killer (NK) cells	MHC-independent recognition of tumor cells	Inhibition through TGF-β and IL-10, Overexpression of CA-125	NK-CAR therapy	(48, 49)
Dendritic cells (DC)	MHC- dependent recognition of tumors and antigen presentation	Inhibition of DC maturation by VEGF and IL-10	Neoantigen-based DC vaccines	(49)
Neutrophils	Pro-tumor	Inhibits proliferation of CTLs via complement	Anti CXCR1/2 therapy	(21, 23)

The table summarizes the modes of inhibition of the immune function in the OC cellular TME and the scope for immunotherapies to target them.

The abundance of Tregs in the OC-TME could also contribute to tumor immune privilege and reduced patient survival in OC. Tregs constitute 10 to 17% of T cells in the OC ascites microenvironment and they suppress the anti-tumor CTL effector responses (26). Tumor cells and TAMs recruit CCR4+ Tregs to the TME via secretion of chemokine CCL22. The CCR8-CCL1 and CCR8-CCL18 axis also plays a major role in the migration and infiltration of CD4^+^CCR8^+^ Tregs into ovarian tumor tissues that significantly overexpress CCL1 and CCL18 ligands (S. 27). Blocking these receptors on Tregs can potentially impede Treg cell infiltration into the TME and thus help augment CTL functions.

Targeting the aforementioned suppressive cells of OC TME to ameliorate immunosuppression has been a focus of numerous studies (**Table 1**).

## Immunotherapies in OC

Various immunotherapies including checkpoint blockade, CAR-based therapies, and tumor vaccines are currently being tested for OC. However, the success has been mixed thus far as described below:

Several clinical trials on the use of immune checkpoint inhibitors (ICIs) in the treatment of ovarian carcinoma are underway. The PD-1/PD-L1 axis diminishes T-cell activity (28), making PD-1 a promising target for immunotherapy in several cancers (29, 30). In addition, immunosuppressive cells such as Tregs express high levels of PD-1 contributing to the suppressive TME (J.-H. 31, 32). However, anti-PD-1/PD-L1 monotherapy in OC has only shown a modest overall response rate of 9-22% (33). An objective response rate study, KEYNOTE 100, evaluated Pembrolizumab (anti-PD1 antibody) as a single treatment agent in recurrent ovarian carcinoma, eventually reporting a modest response (34). Another trial, JAVELIN Ovarian 100, evaluated Avelumab (anti-PD-L1) in combination with and/or following platinum-based chemotherapy in advanced ovarian carcinoma and reported a marginally promising response, concluding the failure of Avelumab as an individual treatment entity (35). Unfortunately, 95% of the patients in the Ipilimumab monotherapy (NCT 01611558) failed to complete the phase II trial due to toxicity or death (36). A non-randomized interventional clinical trial with a small subset of patients is ongoing which is investigating the efficacy of anti-PD-1, anti-CTLA4, and LAG3 antibodies in metastatic OC patients (37).

The use of combination therapy of Ipilimumab (anti-CTLA4), Nivolumab (anti-PD-1), and Avelumab along with carboplatin/doxorubicin respectively still showed low improvement in progression-free survival (35, 50, 51). Together, while these results uncovered the potential of combination immunotherapies in OC, it is apparent that deeper research into both the choice of checkpoint therapy targets as well as the non-immune targets for more effective chemo-immunotherapies is warranted.

Multicomponent therapies targeting both cancer-derived factors as well as T-cell inhibitory receptors hold great promise in OC. For example, PARP inhibitors worked well in an HR-deficient background by exhibiting synthetic lethality and selective toxicity in OC (52, 53). The combination of PARP inhibitor (Niraparib) with anti-PD-1 (Pembrolizumab) showed a 73% disease control rate in pre-clinical study models of high-grade serous OC (54) An active phase III trial involving the use of Rucaparib (PARP inhibitor) and Nivolumab is currently ongoing (55) and results are awaited. In addition, VEGF-A is an interesting target because not only it is pro-angiogenic but is also known to increase the expression of PD-1 on T-cells and induce proliferation of Tregs, all of which add to the immunosuppressive TME. A combination of anti-PD-1 with Bevacizumab (anti-VEGF) reported a clinical benefit of about 95% in recurrent OC patients with a progression-free survival (PFS) of over 12 months (56). A phase III interventional trial involving 1404 patients administered with Bevacizumab, PARP inhibitor, and anti-PD-1 is ongoing which could lead to promising treatment options for advanced-stage OC patients (57). Some of the other immunotherapy trials involving anti-VEGF treatments are listed in **Table 2**.

**Table 2:** Immunotherapy-based clinical trials in OC.

Sl	Trial	Year	Intervention	Subjects	Phase	Target	Findings/Status	Link
1	AURELIA	2016	Bevacizumab, Liposomal Doxorubicin, Baclitaxel, Topotecan	361	III	VEGF	Extended median PFS from 3.4 months to 6.7 months and no benefit to overall survival (OS)	https://clinicaltrials.gov/ct2/show/record/NCT00976911
2	GOG-0213 (NCT00565851)	2016	Bevacizumab, Carboplatin, Docetaxel, Gemcitabine Hydrochloride, Paclitaxel	1052	III	VEGF	Improvement of OS (42.2 vs. 37.3 months (mo), with bevacizumab plus carboplatin and paclitaxel, followed by bevacizumab maintenance	https://clinicaltrials.gov/ct2/show/NCT00565851
3	OCEANS (NCT00434642)	2016	Carboplatin, Gemcitabine, Bevacizumab	484	III	VEGF	Improved PFS (12.4 vs. 8.4 mo, but demonstrated no significant benefit to OS	https://clinicaltrials.gov/ct2/show/NCT00434642
4	GOG-0218 (NCT00262847)	2018	Bevacizumab, Carboplatin, Paclitaxel	1873	III	VEGF	Extended PFS (14.1 vs. 10.3 mo	https://clinicaltrials.gov/ct2/show/NCT00262847
5	NK-CART (NCT03692637)	2018	Anti-Mesothelin CAR NK Cells	30	Early phase I	Anti-Mesothelin CAR NK Cells	NA	https://www.clinicaltrials.gov/ct2/show/NCT03692637
6	V3-OVA	2018	Tableted vaccine (V3-OVA) containing ovarian cancer antigens	20	II	ovarian cancer antigens	Recruiting	https://clinicaltrials.gov/ct2/show/NCT03556566
7	Vaccines	2008	Biological: DC vaccination	36	II	DC vaccination with tumor lysate or WT1 and MUC1 peptide	Recruiting	https://clinicaltrials.gov/ct2/show/NCT00703105
8	Vaccine	2022	DC vaccine	09	I	Dendritic Cell Vaccination With Standard Postoperative Chemotherapy	Active	https://clinicaltrials.gov/ct2/show/NCT05270720
9	Anti-PD-1	2021	BGB-A317+ albumin-bound paclitaxel, Carboplatin	40	II	Humanized IgG4 monoclonal antibody with high affinity/specificity for PD-1	Recruiting	https://clinicaltrials.gov/ct2/show/NCT04815408
10	Anti-CD40 CDX-1140	2023	Anti-CD40 Agonist Monoclonal Antibody CDX-1140 Bevacizumab, Pembrolizumab	80	II	Pembrolizumab combined with Bevacizumab with or without agonist anti-CD40 CDX-1140	Active	https://clinicaltrials.gov/ct2/show/NCT05231122
11	Complement C3 (NCT04919629)	2021	Inhibition of complement C3 + anti-PD-1	40	II	Complement C3 with Pembrolizumab	Recruiting	http://clinicaltrials.gov/ct2/show/NCT04919629

A summary of some of the immunotherapy-based clinical trials in OC describing the immunotherapeutic target, intervention strategy, and patients enrolled in the trials.

Adoptive immunotherapy has emerged as one of the most popular immunotherapeutic approaches for advanced cancers. Several studies utilizing lymphocytes expressing chimeric antigen receptors (CARs) specific to overexpressed antigens in OC are currently underway. The administration of autologous anti-mesothelin CAR-T cells showed an increased immune response in recurrent OC patients (58). Trials are also ongoing for folate receptor and MUC-16-based CAR-T cell therapy (59–61). In addition to CAR-T, the research into NK-CAR therapies is also gaining momentum for OC immunotherapy. The immunosuppressive microenvironment of OC including the malignant ascites suppresses the expression of NK cell receptor NKp30 which leads to reduced IFN-γ production and NK cell activation. Patient-derived experimental models have reported reduced tumor migration and increased survival benefits in the presence of NK cells (48, 62). Trials targeting the NKG2A inhibitory ligand- commonly expressed on both NK and CD8+ T-cells-using monalizumab in OC, unfortunately, showed a non-significant clinical effect (63). A clinical trial based on the NK-CARs targeting mesothelin in OC is currently underway (64).

Dendritic cells (DC) present tumor antigens to T-cells. DC-based vaccine strategy holds considerable promise in OC. There are about 126 registered OC vaccine trials, of which 24 are based on DC vaccines (65, 66). A small phase I trial with 11 patients who received DC loaded with HER-2 derived peptide reported an overall 3-year survival rate of 90% (67). Furthermore, a DC vaccine with folate receptor-α peptide in advanced OC demonstrated a PFS of 39% without any grade 3 toxicity (68). DC-based therapeutic strategies hold great promise in OC because the OC TME shows increased DC infiltration. Although the infiltrated DC possess weak antigen-presenting ability due to decreased expression of MHC-II and co-stimulatory molecules (69–71), they still offer attractive potential for improved immunotherapies for alleviating the suppressive effect of TME on antigen presentation pathways (**Table 2**).

## Challenges in effective immunotherapy response

Multiple features of OC-TME limit successful immunotherapy response by reinforcing the immunosuppressive TME. The diverse cellular and soluble factors of the primary TME and the malignant ascites pose a significant challenge to designing immunotherapeutic strategies for OC. Understanding the intricate super-and sub-cellular networks that generate and maintain the immunosuppressive microenvironment in both TMEs is crucial for developing targeted immunotherapies with higher efficacy and safety. For example, CCL22 and TGF- β produced by the cancer cells and cancer-associated fibroblasts could recruit Tregs which in turn produce TGF-β and induce the conversion of naïve CD4^+^ T cells into Tregs (39, 72). Furthermore, the highly glycolytic OC-TME contains lactic acid that drives the metabolism and proliferation of T-regs contributing to a highly immunosuppressive microenvironment (40, 73). The suppressive cells such as MDSCs and TANs are found not just in the primary tumor but also in the peripheral blood and ascites of OC patients and are significantly associated with a shorter survival rate (42). MDSCs secrete arginase-1 which lowers the levels of arginine, an essential amino acid required for effector T-cell differentiation and NK cell survival. Additionally, nitric acid produced by MDSCs activates STAT-1 and downregulates the IFN-γ expression in NK and cytotoxic T-cells. MDSCs also upregulate the expression of PD-L1 leading up to T cell exhaustion (74). The cancer cells secrete M2-type cytokines such as IL-10, VEGF, PDGF, CXCL12, CCL2, and CCL3 that mediate the recruitment of monocytes and M0 macrophages and drive them towards an M2 pro-tumor phenotype observed often in OC tissues (41, 47). M2 macrophages or tumor-associated macrophages (TAMs) secrete CCL22 and draw Tregs and MDSCs into the TME strengthening an immunosuppressive milieu. This is further aided by mature granulocytic neutrophils that acquire an inhibitory phenotype on getting recruited to the ascites microenvironment and suppress the immune cells via complement mediated pathway (10). The OC tumor cells also express checkpoint ligands such as PD-L1 and B7-H4 which drives the inactivation of cytotoxic T cells (26). In addition, OC is considered to be a cold tumor with low median tumor mutational burden at 3.6 mutations/million bases (75). The relatively low mutational burden hinders the identification of neoantigens by immune cells impeding the intended anti-tumor immune response (1, 76).

OC metastasis involves the generation and shedding of malignant tumor spheroids in the peritoneal cavity. These spheroids are unique to OC and are highly specialized entities that pose an additional barrier to successful immunotherapies owing to their distinct metabolic reprogramming compared to the primary tumor and lack of vascularization. The spheroids also play a role in ECM remodeling, the release of immunosuppressive cytokines (IL-6, IL-8)(8), growth factors (VEGF)(77), and immunosuppressive molecules (CA125, Galectins, LPA) that limit effector immune cell infiltration and function (8, 78, 79). Characterizing and targeting the spheroid metabolic vulnerabilities along with enhanced drug delivery strategies to compensate for the lack of vascular supply as well as overcoming the ECM barrier around the spheroids should be integrated into immunotherapies to efficiently break immune suppression and clear tumor burden.

## Discussion

Immunotherapy has emerged as a powerful strategy for the treatment of numerous types of cancers, and its application in OC has yielded encouraging results thus far. However, the results are still far from perfect when compared to other malignancies, and the success rate is rather limited. Of the immunotherapy trials conducted yet, the multi-pronged therapeutic approaches targeting multiple aspects of immunosuppression as well as the tumor-derived factors have generated the most promising results and should be built upon to generate more effective and safer therapies. One overlooked aspect of OC immunotherapy is the mechanical heterogeneity that exists within tumor cells that potently changes the T cell immune response (80, 81). In addition, there is substantial deposition of extracellular matrix as well as soluble glycoproteins produced by the tumor cells that impede optimal immune response in OC (82–84). Interaction of cancer cells with collagen results in inhibition of apoptosis of the cancer cells, promotes epithelial-to-mesenchymal transitions and eventually results in chemoresistance (85, 86). As a result, the cancer cells escape peritoneal immunosurveillance, an evasion process further aided by complex intercellular crosstalk between the tumor and immune cells within the malignant ascites (12). The ECM components also inhibit immune infiltration as well as the effector function of immune cells at the tumor site by directly providing cell adhesion and immunomodulatory ligands. Some of these receptors such as DDR-1, DDR-2, and LIAR-1 have been identified and targeted in this regard, improving survival outcomes (87).

A major challenge in immunotherapies that is not unique to OC is stratifying patients who would benefit from immunotherapy. Biomarker identification can serve as a cornerstone in this regard. The MA is rich in cytokines (IL-6, IL-10, VEGF)(15, 88, 89), DAMPs (damage-associated molecular patterns) (10) and other immune-modulatory factors (complement proteins, fibronectin, α-1 acid) (90) that can be used as predictive tools for selective immunotherapeutic strategies. Another biomarker in OC patients is alterations in extracellular matrix protein collagen. For instance, excessive collagen type XI alpha 1 (COL11A1) secretion in OC is involved in enhancing cell invasiveness and tumor formation by activating matrix metalloproteinases (MMP). siRNA-mediated silencing of COL11A1 decreases tumor formation and lung colonization. Targeting COLL11A1 or its effector MMP3 can thus be a viable treatment option in OC (13). Interaction of cancer cells with collagen also results in the inhibition of apoptosis of the cancer cells, promotes epithelial-to-mesenchymal transitions, and eventually results in chemoresistance (85, 86). Homologous DNA repair deficiency is another important biomarker in OC (91). Poly (ADP-ribose) Polymerase (PARP) recruits the homologous DNA repair machinery and prevents the activation of the non-homologous end-joining pathway (91). PARP has also been an attractive target in the recent OC immunotherapy trials. In addition to previously identified biomarkers for OC, new biomarkers are being identified in the malignant ascites that could also serve not only as a prognostic tool but also as a target for designing novel immunotherapies (92). The CD4+/CD8+ ratio is also used as a prognostic marker for monitoring the effectiveness of immunotherapies in OC, and a high CD4+/CD8+ ratio is associated with poor clinical outcomes in OC patients (93, 94). The combination of existing predictive biomarkers such as expression of PD-L1 (34, 95), increased infiltration of CD8^+^ T cells (M. 96, 97) and increased IFN-γ levels in serum (98), increased expression of CA125 (99), VEGFR3 (98), TGF-β (100) would greatly help with clinical outcome and thereby help in identification of immunotherapy responder population. Importantly, although most of the current immunotherapy strategies are being assessed in the recurrent OC scenario, careful identification of response-associated predictive signatures in patients in the primary treatment stages itself could be immensely beneficial in tackling recurrent OC.

Overall, multicomponent immunotherapies are shaping a new era of ovarian cancer treatment. A deeper understanding of tumor–immune crosstalk, the discovery of more OC-specific checkpoint mechanisms, more specific strategies to target metastatic spheroids, and advanced technologies for early detection as well as patients’ stratification during therapy would bolster the efficacy and safety of immunotherapeutic approaches leading to more effective outcomes.

## Data availability statement

The original contributions presented in the study are included in the article/supplementary material. Further inquiries can be directed to the corresponding author.

## Author contributions

SaK: Writing – original draft, Writing – review & editing. SA: Writing – review & editing. MK: Visualization, Writing – original draft. PB: Writing – review & editing. SuK: Conceptualization, Writing – original draft, Writing – review & editing.
